# Evidence from a large-scale meta-analysis indicates eczema reduces the incidence of glioma

**DOI:** 10.18632/oncotarget.11545

**Published:** 2016-08-23

**Authors:** Guannan Wang, Suling Xu, Chao Cao, Jing Dong, Yudong Chu, Guijuan He, Zhiwei Xu

**Affiliations:** ^1^ Academy of Nursing, Zhejiang Chinese Medical University, Hangzhou 310053, China; ^2^ Blood Purification Center, Ningbo Medical Center, Lihuili Hospital, Ningbo University, Ningbo 315041, China; ^3^ Department of Dermatology, Affiliated Hospital of Medical College, Ningbo University, Ningbo 315020, China; ^4^ Department of Dermatology, Ningbo First Hospital, Ningbo University, Ningbo 315010, China; ^5^ Department of Respiratory Medicine, Ningbo First Hospital, Ningbo University, Ningbo 315010, China; ^6^ Department of Nephrology, Ningbo Medical Center, Lihuili Eastern Hospital, Ningbo 315040, China; ^7^ Department of Critical Care Medicine, Ningbo Medical Center, Lihuili Eastern Hospital, Ningbo 315040, China

**Keywords:** glioma, eczema, meta-analysis

## Abstract

We investigated the relationship between eczema and the risk of primary glioma. Relevant studies were selected through electronic searches of PubMed and EMBASE. A meta-analysis of 12 case-control studies and one cohort study was performed. A fixed effect model was applied to analyze 13 studies consisting of 10,897 glioma cases and 56,081 controls. Odds ratios (ORs) and 95% confidence intervals (CIs) were calculated to assess the strength of the associations. The data demonstrate that eczema significantly reduces the risk of glioma (OR = 0.69, 95% CI = 0.61–0.78, *P* < 0.001). Additional studies with larger patient cohorts are required to validate our findings.

## INTRODUCTION

Glioma is the most common type of brain tumor worldwide. It affects approximately six per 100,000 individuals annually [1–3]. The prognosis of glioma patients is poor. The 5-year relative survival rate is approximately 25% [3]. Several risk factors for glioma have been identified. For example, exposure to high-dose ionizing radiation was shown to increase the risk of glioma [4]. Additionally, nervous system dysfunction was shown to contribute to glioma development [5–8]. Several rare genetic syndromes have also been associated with an increased risk of glioma. Relatives of individuals with glioma accounted for two-fold more cases than non-relatives [9, 10]. Finally, allergic disorders such as asthma, eczema, and hay fever have been associated with a decreased risk of glioma [11–14].

Atopic dermatitis, or eczema, is common skin condition worldwide [15]. Patients with atopic eczema have high concentrations of circulating allergen-specific immunoglobulin E (IgE) antibodies. The disease course involves an initial Th2 phase that precedes a chronic stage in which Th0 and Th1 cells are the predominant cell types [16, 17]. Previous studies have provided evidence that allergic conditions are associated with glioma. The heightened immune response in patients with atopic conditions may contribute to cancer development [18]. Evidence from animal studies has also supported an association between biomarkers of allergic diseases and a reduced incidence of glioma [19]. However, the inverse associations described may have resulted from bias in the methodology used in the epidemiological studies [17].

Only 0.05%–4.7% of glioma patients survive for 5 years after diagnosis [20]. Previous studies have indicated that eczema is associated with a reduced incidence of glioma. We performed a meta-analysis to investigate the potential association between eczema and risk of glioma.

## RESULTS

**Figure 1:**
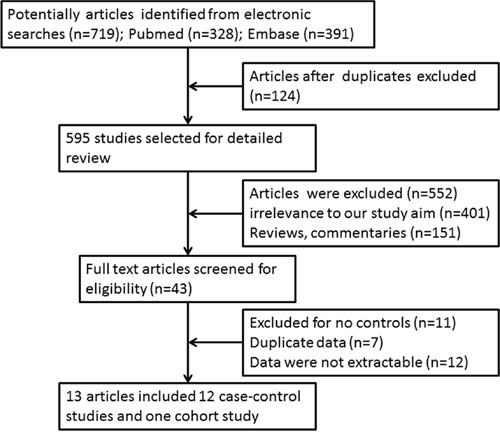
Literature survey and study selection

The literature selection process is shown in Figure 1. A total of 719 entries were identified in our search. After an initial assessed of the study titles and abstracts, 43 articles remained for analysis. We excluded 12 studies because the data could not be extracted. Four studies were part of an international analysis [21–24] and included the largest sample sizes [23]. Three case-control studies had partially overlapping subjects [25–27]. In this case, the study with the largest sample size was selected [27]. Finally, 13 studies (12 case-control studies [13, 23, 26–36] and one cohort study [25]) met the eligibility criteria.

The author information, publication years, numbers of control and glioma cases, odds ratios (ORs), and 95% confidence intervals (CIs) for glioma in individuals with eczema for the studies that were included in the analysis are shown in Table 1. Thirteen of the studies were published between 1999 and 2015. These studies consisted of 10,897 glioma cases and 56,081 controls. The Newcastle-Ottawa Scale (NOS) was used to assess the quality of the studies. The scores (on a scale of 0–9) ranged from 6–8 (moderate to high quality) for all studies, with an average score of 6.9. The scores are shown in Table 2.

**Table 1:** Description of the epidemiological studies that assessed the association between eczema and the risk of glioma

Study (Publication year)	Case	Control	Country of Cases	Design	Exposure assessment	Possible confounders adjusted for in analysis	Cases of diagnostic criteria	OR
Schlehofer (1999)	1178	1987	AUS, CAN, FRA, GER, SWE, USA	case-control	face-to-face interviews; Self-administered questionnaires	age, gender and region	histologically	0.64 (0.47-0.86)
Brenner (2002)	782	799	USA	case-control	proxy interviews; questions inquiring	age, sex, race or ethnicity and distance of residence	histologically	0.76 (0.45-1.27)
Schwartzbaum (2003)	42	29573	SWE	cohort	questionnaire mailed	same gender twins born	histologically	0.52 (0.19-1.48)
Schoemaker (2006)	965	1716	GBR	case-control	a computer-assisted interviews	age, sex and geographical distribution	histologically	0.74 (0.56-0.97)
Harding (2007)	575	6292	GBR	case–control	questionnaire	age, sex and region	histologically	0.89 (0.65-1.21)
Wigertz (2007)	1527	3309	DEN, FIN, NOR, SWE, UK	case-control	interviews; proxy interviews	age, sex, and geographic region	histologically	0.70 (0.61-0.80)
Scheurer (2008)	325	600	USA	case-control	questionnaires; show cards	age, gender, ethnicity, and location	confirmed by a neuropathologist	0.34 (0.23-0.51)
Berg-Beckhoff (2009)	366	1494	GEM	case-control	personal interviews (CAPI)	age, sex and study region	histologically	0.91 (0.65-1.27)
Il'yasova (2009)	388	448	USA	case-control	web-based;telephone survey	age, gender, and race/ethnicity	histologically	0.52 (0.28-0.98)
Lachance (2011)	855	1160	USA	case-control	questionnaires	age, gender, ethnicity, and residence	pathologically	0.62 (0.51-0.76)
Safaeian (2013)	851	3977	USA	case-control	questionnaires	age, gender, broad categories of race and ethnicity	histologically	0.83 (0.58-1.19)
Turner (2013)	793	2520	CAN	case-control	a computer-assisted personal interview questionnaire	age, sex, region, and country of birth	histologically	0.67 (0.49-0.91)
Krishnamachari (2015)	2250	2206	USA	case-control	proxy; selfreport	age, race, and gender	histologically	0.90 (0.50-1.40)

**Table 2:** Assessment of the methodological quality of the case-control and cohort studies based on the Newcastle-Ottawa Scale

First author, (year)	Selection				Comparability	Outcome			Overall Quality^b^
*case–control studies*	*Definition of cases*	*Representativeness of cases*	*Selection of controls*	*Definition of controls*	*Comparability the design or analysis*	*Ascertainment of exposure*	*Same method for cases and controls*	*Non-Response rate^a^*	
Schlehofer (1999)	1	1	1	1	2	0	1	0	7
Brenner (2002)	1	1	0	1	2	0	1	0	6
Schoemaker (2006)	1	1	1	1	2	0	1	0	7
Harding (2007)	1	1	1	1	2	0	1	0	7
Wigertz (2007)	1	1	1	1	2	0	1	0	7
Scheurer (2008)	1	1	1	1	2	1	1	0	8
Berg-Beckhoff (2009)	1	1	1	1	2	0	1	0	7
Il'yasova (2009)	1	1	0	1	2	0	1	0	6
Lachance (2011)	1	1	1	1	2	0	1	0	7
Safaeian (2013)	1	1	1	1	2	0	1	0	7
Turner (2013)	1	1	1	1	2	0	1	0	7
Krishnamachari (2015)	1	1	1	1	2	0	1	0	7
*cohort studies*	*Representativeness of the exposed cohort*	*Selection of the non-exposed cohort*	*Ascertainment of exposure*	*Outcome of interest was not present at start of study*	*Based on the design or analysis*	*Assessment of outcome*	*Follow-up long enough for outcomes to occur*	*Adequacy of follow-up of cohorts*	
Schwartzbaum (2003)	0	1	0	1	2	1	1	1	7

a When there was no significant difference in the response rate between both groups by using a chi-squared test (P > 0.05), one point was awarded.

b Total score was calculated by adding up the points awarded in each item.

**Figure 2:**
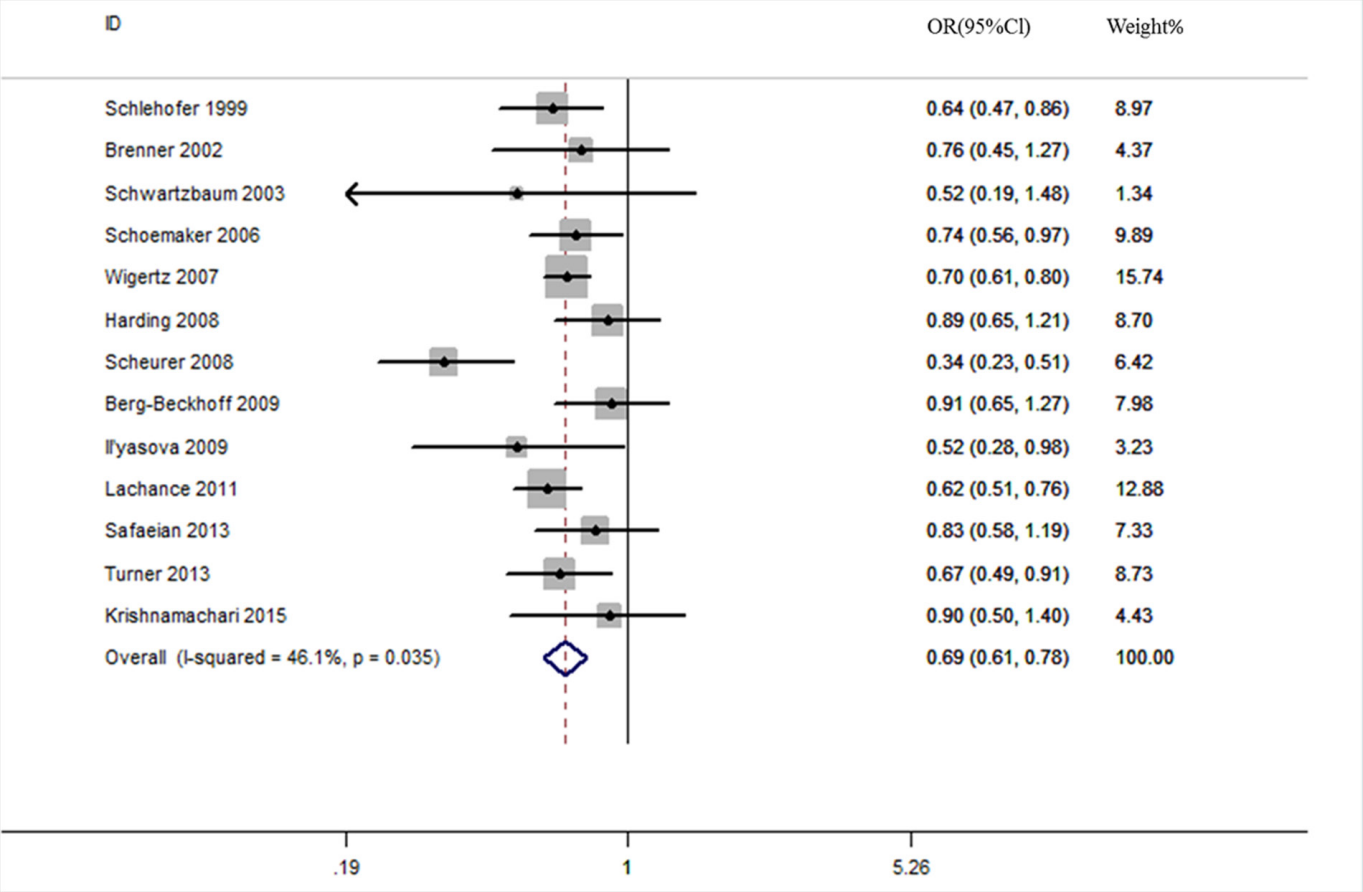
Eczema is associated with a reduced risk of glioma

**Figure 3:**
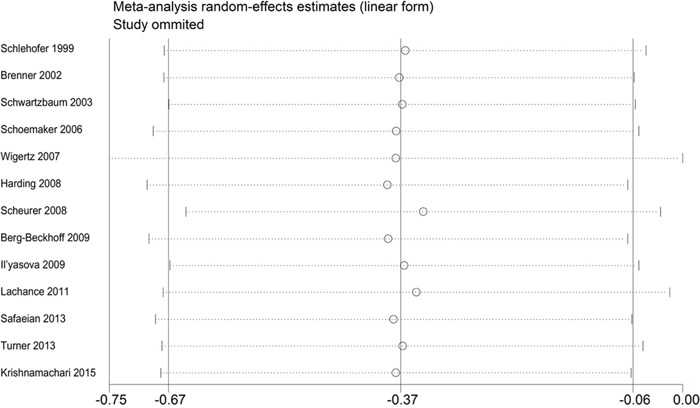
Sensitivity analysis of the association between eczema and risk of glioma

**Figure 4:**
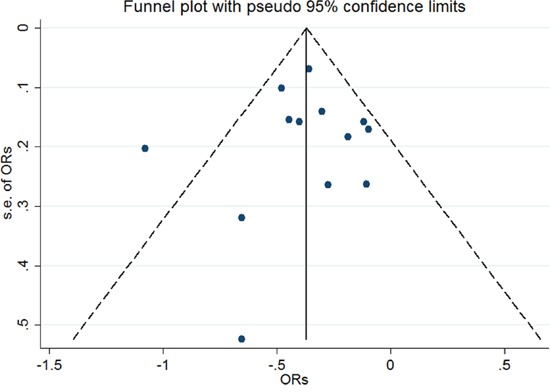
Funnel plot of the included studies (n = 13) to evaluate publication bias

An assessment of heterogeneity suggested that the 13 studies included in the analysis were homogeneous (Chi^2^ = 22.25, *I^2^*** =46.1 %, *P* = 0.035). Therefore, a fixed-effects model was selected in order to calculate the ORs. In the pooled analysis, individuals with a history of eczema had a decreased risk of incident glioma (OR = 0.69, 95% CI = 0.61–0.78, *P* < 0.001; Figure 2). In the sensitivity analysis, we removed one single study each time from the overall analysis and evaluated the influence of the omitted data. When any part of the study was omitted, the pooled ORs and 95% CIs did not significantly change (Figure 3). These data indicated that no single study impacted the overall ORs. The shapes of the funnel plots did not show obvious asymmetry (Figure 4). We also performed Egger's linear regression test and demonstrated that there was no publication bias in the meta-analysis (*P* = 0.84).

## DISCUSSION

Previous studies have shown that eczema is associated with a reduced risk of various malignances (e.g. skin, lung, and cervical) [17, 37, 38]. However, the relationship between eczema and risk of glioma has not been fully elucidated. We performed a meta-analysis based of 13 studies consisting of 10,897 glioma cases and 56,081 controls to investigate the potential relationship between eczema and glioma susceptibility.

The results from our study revealed an inverse association between eczema and risk of glioma (OR = 0.69, 95% CI = 0.61–0.78, *P* < 0.001), suggesting that eczema may modify the risk of glioma through modulating the immune system. Our results are consistent with those of previous studies, which demonstrated an association between allergies and glioma [14, 39]. Linos et al. [14] found that allergic conditions were associated with a reduced risk of glioma (OR = 0.61, 95% CI = 0.55–0.67) compared to controls. Additionally, Chen et al. [39] determined that the pooled OR for individuals with a history of allergic conditions was associated with a significant risk reduction (OR = 0.69, 95% CI = 0.52–0.69, *P* < 0.001) based on a meta-analysis of 12 observational studies. However, the sample size was not sufficient to detect the effect of eczema on the risk of glioma. In our meta-analysis, we systematically evaluated existing data and provided more credible evidence for an association between eczema and glioma risk.

Our data indicate that eczema significantly reduces the risk of glioma. This inverse association may result from reverse causality. Because the tumor itself can suppress the immune system [40], glioma patients often have immunological defects such as a delayed hypersensitivity response and reduced number of circulating T cells [41]. The tumour would be able to effect the immune system in its preclinical phase. However, the exact schedule can vary. In general, this effect develops relatively slowly with low-grade gliomas, but quickly with high-grade gliomas. Most previous studies on the relation between glioma risk and eczema were case-control studies, and so exposure was assessed when the tumour was already present. These findings are supported by data from Schoemaker et al. [36], who found that the risk of glioma was reduced in patients who reported having eczema 1 year prior to glioma diagnosis 0.61 (95% CI: 0.43–0.88). This was not observed in patients who did not have eczema within a year of glioma diagnosis (OR 5 0.94, 95% CI: 0.63–1.39) [36]. An inverse correlation was also observed in patients who were diagnosed with eczema at an early age or many years prior to diagnosis with glioma [36]. However, reverse causation dose not explain the overall findings, and most studies of the history and timing of eczema had insufficient data.

The mechanisms underlying the relationship between eczema and glioma have not been fully elucidated. Eczema was previously associated with increased eosinophil and IgE levels. Recently, several studies have reported that eosinophilia may prevent cancer development [18, 42, 43]. Eosinophils have been observed in the solid tumor microenvironment, and may be involved in immune surveillance and the antitumor immune response [18, 44]. Adults with glioma were found to have lower antibody titers to varicella zoster virus and reported fewer chicken pox infections than controls, suggesting the immune response to prior infections could protect against glioma development [45]. The IgE level may be a better marker of an association between allergies and glioma. IgE is produced and regulated by B, T helper type 2 (Th2), and T helper type 17 (Th17) cells. We previously found that germ line polymorphisms increased the risk of lung cancer [46]. SNPs and haplotypes in genes encoding IL-4, IL-4R, and IL-13 have been shown to play a critical role in allergy and IgE production [47]. Several studies have reported that SNPs in IL-4, IL-4R, and IL-13 were inversely correlated with the incidence of glioma [26, 48, 49]. New glioma treatments have been developed based on these discoveries.

This is the first meta-analysis to demonstrate an association between eczema and risk of glioma. Our analysis had several advantages. First, we included 13 studies consisting of 10,897 glioma cases and 56,081 controls. Second, we included only case-control or cohort studies. Almost all of the cases in the included studies were histologically confirmed with well matched controls. Third, there was negligible heterogeneity (*I^2^*** = 46.1%, P = 0.035). Finally, the studies were moderate to high quality according to the NOS.

Our meta-analysis also had several limitations. First, the data were derived from observational studies subject to inherent biases such as unmeasured and uncontrolled confounders. Second, there were differences in study design. We included 12 case-control and one cohort study in our analysis. Different methods were also used in the exposure assessments. In most studies, interviews with questionnaires were used to obtain information regarding participant history of eczema. Some studies used proxy interviews while others relied on self-reported data. The study methods also differed and included telephone-, computer-, and web-based methods as well as face-to-face interviews conducted by trained professionals. The exposure assessment was based on patient memory. However, it is possible that cognition and memory could have been impaired in the glioma patients, which could have affected the results. In order to avoid these biases, several studies evaluated the quality of the patient interviews [27].

In summary, our meta-analysis has demonstrated that eczema is associated with a reduced risk of glioma. Given that individuals with a history of eczema may have enhanced antitumor immune surveillance, patients who have a high risk of glioma could be screen based on history of eczema. Our results may assist in glioma prevention, early diagnosis, and treatment. Additional studies are warranted to elucidate the biological mechanisms by which eczema reduces the risk of glioma.

## MATERIALS AND METHODS

### Search methods

We identified studies that were published between 1979 and 2015 using PubMed and EMBASE. The search terms included the following: brain tumor OR glioma OR CNS tumors OR central nervous system tumors AND allergy OR atopy OR eczema. Studies were not excluded based on language. We first scanned the titles and abstracts to identify relevant papers. We then searched for related articles that were potentially relevant. We also reviewed the references in all identified publications to identify additional studies. We aimed to include all published case-control and cohort studies that reported a correlation between eczema and glioma in the meta-analysis. If important values could not be extracted from the articles, we contacted the corresponding author to obtain the missing data, details regarding additional studies, and the data for confounding factors.

### Inclusion/exclusion criteria

The inclusion criteria were the following: (1) case–control or cohort study that investigated the association between eczema and glioma; (2) available patient data including the histological or clinical diagnosis (a diagnosis of eczema was accepted based on clinical criteria); (3) used OR or relative risk (RR) with CI to estimate the risk of glioma in individuals with a history of eczema compared to controls without the disease; and (4) recently published data in cases involving more than one published study of the same population. If studies had partly overlapping subjects, the study with the larger sample size was selected. The literature review and data extraction were independently performed by two investigators. Differences in opinion were resolved by discussion.

### Data extraction and quality assessment

Two authors independently extracted the data from each eligible study. The data included: (1) the name of the first author and affiliation; (2) publication year; (3) sample size; (4) nationality of the patients; (5) study design; (6) exposure assessment; (7) adjustment for potential confounders; (8) diagnostic criteria; and (9) reported outcome measures including OR or RR and 95% CI. Study quality was assessed by two investigators according to the NOS [50]. This scale reflected three parameters: the selection of the study group (4 point scale), the comparability of the groups (2 point scale), and the exposure or outcome of interest (3 point scale). We defined scores of 0–3, 4–6, and 7–9 as low, moderate, and high quality, respectively.

### Statistical analysis

The meta-analysis was performed using the Stata 12.0 program. Cochran's Q-tests were performed to estimate heterogeneity among the individual studies. A *P* < 0.1 was considered significant [51]. The strength of the heterogeneity was measured by the *I^2^*** test, where *I^2^*** = 0 was indicative of absolute consistency and suggested low (25%–49%), moderate (50%–74%), and high (75%) threshold values [52]. A fixed-effect model was selected unless there was heterogeneity, in which case a random-effect model was selected. Sensitivity analysis was performed to evaluate the effect of each individual study on the pooled ORs. Funnel plot symmetry and Egger's linear regression were used to assess publication bias [52, 53]. A P value of 0.05 was considered significant.
